# An autopsy case of anaplastic lymphoma kinase-positive lung cancer exacerbated in a short period of time: a case report

**DOI:** 10.1186/s13256-019-2054-3

**Published:** 2019-04-29

**Authors:** Masahide Takeda, Kazuhiro Sato, Sho Sakamoto, Maya Suzuki, Yuka Izumiya, Naho Kumagai, Kazuhisa Sudo, Yuji Okuda, Mariko Asano, Masaaki Sano, Yasufumi Omori, Katsutoshi Nakayama

**Affiliations:** 1 Department of Respiratory Medicine, Akita University Graduate School of Medicine, 1-1-1 Hondo, Akita, Akita 010-8543 Japan; 2 Department of Molecular and Tumor Pathology, Akita University Graduate School of Medicine, Akita, Japan

**Keywords:** *ALK*-positive lung cancer, *ALK*-positive adenosquamous carcinoma, Alectinib

## Abstract

**Background:**

Anaplastic lymphoma kinase-positive lung cancer is a form of lung cancer that accounts for approximately 5% of non-small cell lung cancers. Recently, anaplastic lymphoma kinase inhibitors have been used for treatment of anaplastic lymphoma kinase-positive lung cancer, and their high clinical effect has also been demonstrated in cases of advanced stage lung cancer. Alectinib is an anaplastic lymphoma kinase inhibitor that it is recognized as a standard drug for primary therapy because of its superiority to crizotinib.

**Case presentation:**

A 37-year-old Japanese man was admitted to our hospital due to multiple brain metastases. An autopsy report revealed that the cause of death was anaplastic lymphoma kinase-positive lung cancer, exacerbated in a short period despite treatment with alectinib. Necropsy revealed anaplastic lymphoma kinase-positive adenosquamous carcinoma of the lung, suggesting that it was involved in the prognosis of this patient. Based on the autopsy results, we reviewed the pathological tissue from transbronchial lung biopsy at the time of clinical diagnosis. The tissue specimen for clinical diagnosis in this case was a papillary adenocarcinoma. However, when this tissue was immunostained, thyroid transcription factor 1-negative and cytokeratin 5/6-positive parts were recognized. This result indicates that we could diagnose this patient as having had adenosquamous carcinoma of the lung.

**Conclusion:**

In cases of anaplastic lymphoma kinase-positive lung cancer poorly responsive to anaplastic lymphoma kinase inhibitors, re-examination of the tissue should be considered because there is a possibility of anaplastic lymphoma kinase-positive adenosquamous carcinoma.

## Background

In recent years, it has been reported that epidermal growth factor receptor (*EGFR*) mutations and the echinoderm microtubule-associated protein-like 4 (*EML4*)-anaplastic lymphoma kinase (*ALK*) fusion gene are involved in the diagnosis of lung cancer, and it is known that the presence or absence of these also affect treatment [1, 2]. *ALK*-positive lung cancer is a form of lung cancer that accounts for approximately 5% of non-small cell lung cancers [3, 4]. Recently, ALK inhibitors have been used for the treatment of *ALK*-positive lung cancer, and their high clinical effect has also been demonstrated in cases of advanced stage lung cancer. Alectinib is an ALK inhibitor that is recognized as a standard drug for primary therapy because of its superiority to crizotinib [5]. We encountered a case in which alectinib was administered as a primary treatment for *ALK*-positive lung cancer with brain metastases, but our patient died in a short period of approximately 4 months. Here, we report on the cause of death revealed at autopsy with a review of the literature.

## Case presentation

**Fig. 1:**
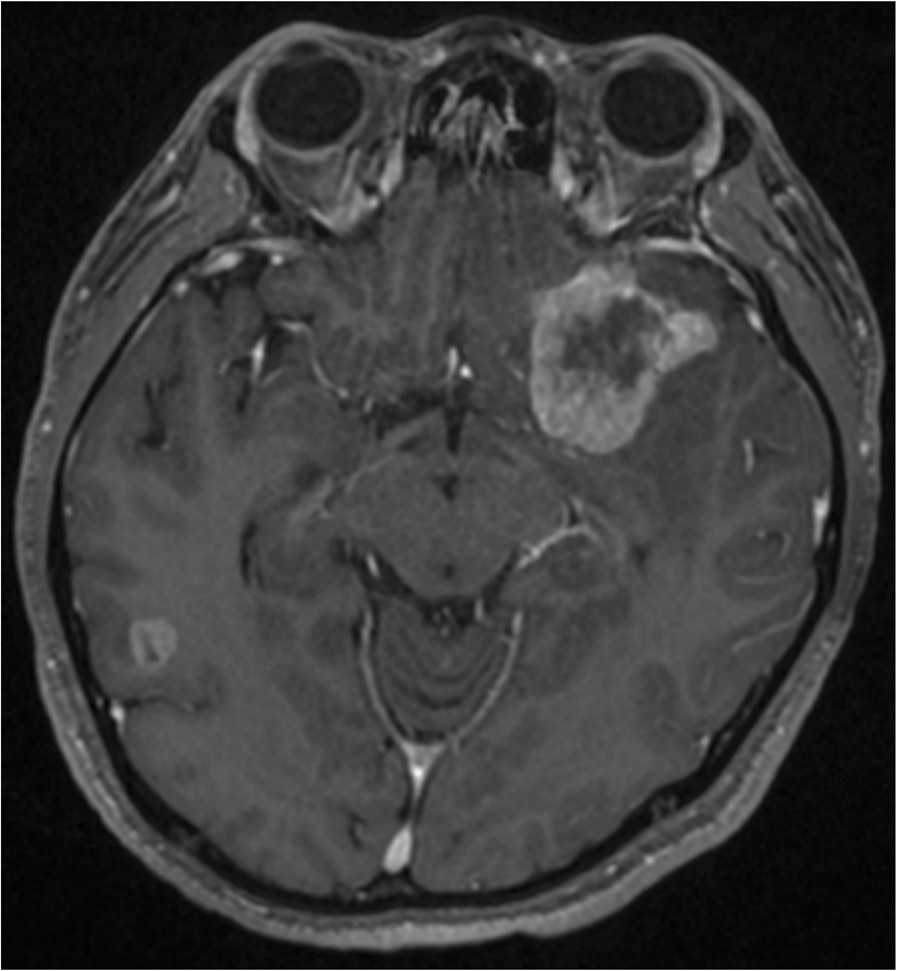
Brain magnetic resonance imaging at first visit to our hospital

**Fig. 2:**
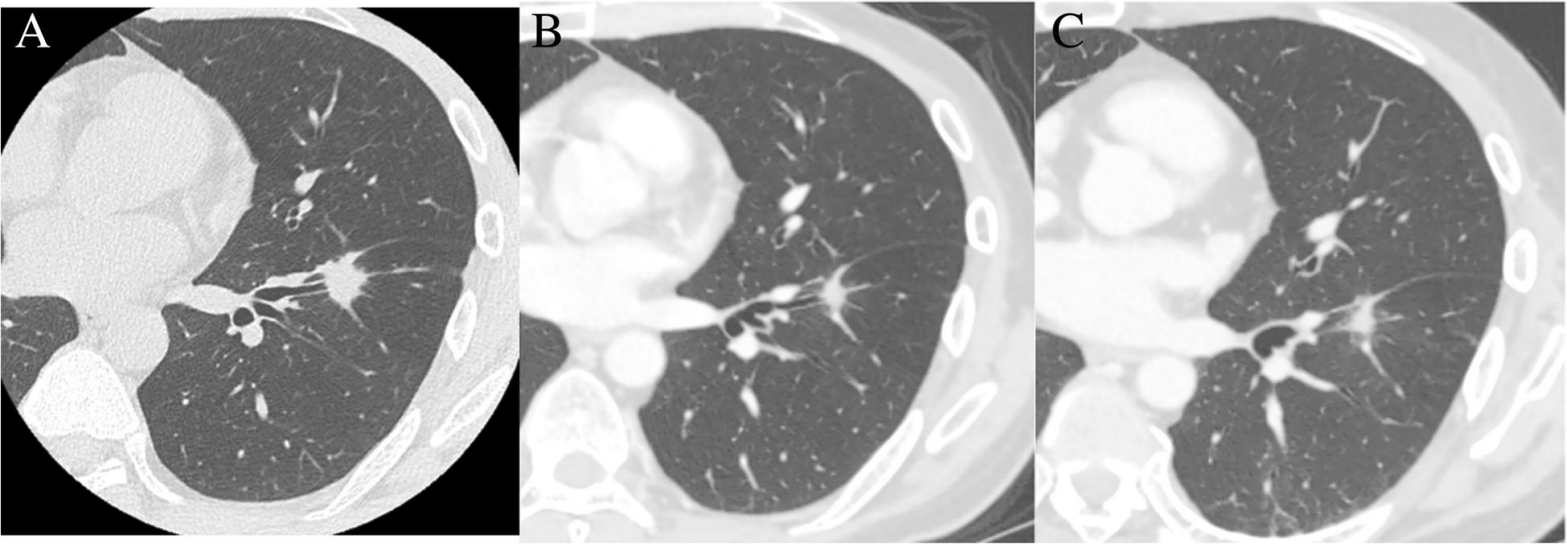
Chest computed tomography at first visit (**a**), 40 days after start of alectinib treatment (**b**), and 90 days after start of alectinib treatment (**c**)

**Fig. 3:**
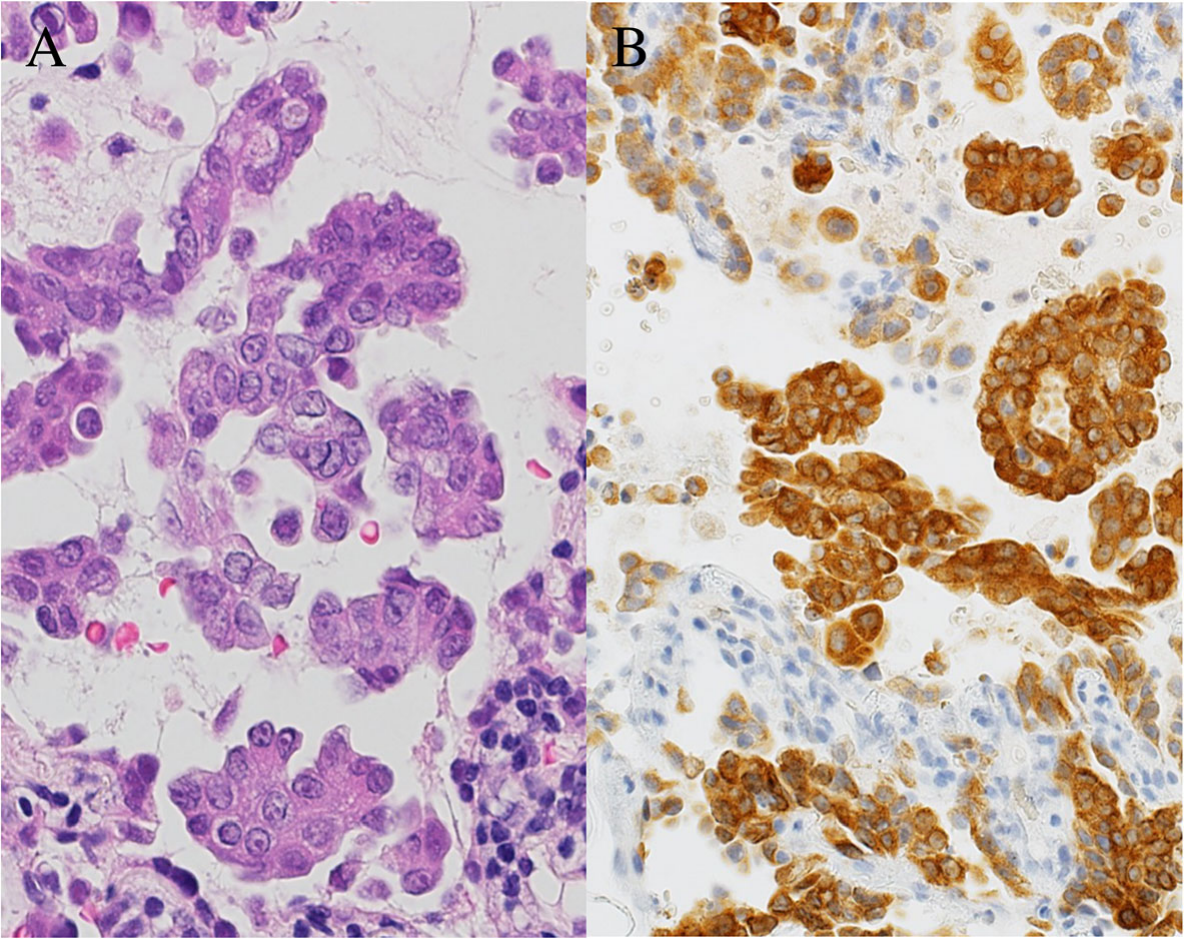
Hematoxylin and eosin stain of bronchoscopy for the middle lobe of the left lung, showing adenocarcinoma cells exhibiting a papillary pattern (**a**). Immunohistochemistry method for anaplastic lymphoma kinase from the same sample (**b**)

A 37-year-old Japanese man was admitted to our hospital due to multiple brain metastases. He was aware of coughing 6 months previously and had a headache 3 weeks ago, so he visited our hospital. Brain magnetic resonance imaging (MRI) revealed multiple brain tumors in the bilateral cerebellum and cerebrum (Fig. 1). Chest computed tomography (CT) showed a 15-mm nodular shadow in the middle lobe of his left lung, and he was referred to our Department of Respiratory Medicine (Fig. 2a). He was admitted for further examination because he was suspected of having lung cancer with brain metastases. There was no special mention in his medical history; there was no alcohol drinking or tobacco smoking history. On physical examination, his body temperature was 36.7 °C, his blood pressure was 122/78 mmHg, his pulse was 56 beats per minute, and his respiratory rate was 12 breaths per minute. His oxygen saturation was 98% in room air. Lung and bronchial sounds were normal. Head, eyes, and nose examinations were unremarkable. His neck had no lymphadenopathy. An examination of his heart, abdomen, and extremities showed no abnormalities. Blood test findings revealed elevation of tumor markers such as carcinoembryonic antigen (CEA) and squamous cell carcinoma antigen (SCC). On day 4 after admission, bronchoscopy was performed. The histology at bronchoscopy for the middle lobe of his left lung is shown in Fig. 3. Adenocarcinoma cells exhibiting a papillary pattern were found, and he was diagnosed as having papillary adenocarcinoma of the left lung according to the World Health Organization (WHO) classification, 4th edition. His adenocarcinoma was positive for *ALK* according to immunohistochemistry and fluorescence *in situ* hybridization (FISH) method (Fig. 3). Based on this observation, he was diagnosed as having *ALK*-positive lung cancer with cerebral metastases, and administration of alectinib 600 mg/day was started from day 23 after admission. Adverse events such as allergic reactions, interstitial pneumonia, and gastrointestinal symptoms were not observed. Pulmonary lesion reduction was confirmed at chest CT on day 37, and he was discharged on day 40 (Fig. 2b). He continued to take orally administered alectinib every day on an out-patient basis. On chest CT at 90 days after initiation of alectinib treatment, continued reduction of the lung lesion and hilar lymph node was confirmed (Fig. 2c). Regarding the brain metastatic lesions, whole brain irradiation (total 30 Gy/15 fractions) was performed from day 9 after admission, and tumor reduction was found on MRI on day 60 after start of alectinib treatment.

Approximately 96 days after start of treatment, he was aware of headaches and nausea, and he visited our emergency department on day 103 of administration. Contrast MRI confirmed the findings of meningeal carcinomatosis. He was re-hospitalized for systemic management, including pain care. Dexamethasone infusion was started for increased intracranial pressure due to meningeal carcinomatosis. His Eastern Cooperative Oncology Group (ECOG) performance status at the time of his second admission was three, which was markedly lower than that at the onset of alectinib administration. He also experienced severe headache pain, so orally administered oxycodone was started. A few days after start of oxycodone administration, it became difficult for him to take orally administered medications, and palliative treatment was started using morphine hydrochloride infusion. Administration of alectinib was discontinued 108 days after start of treatment. Subsequently, it became difficult for him to sit up, his state of consciousness worsened, and his general condition worsened. He died 124 days after the first administration of alectinib.

**Fig. 4:**
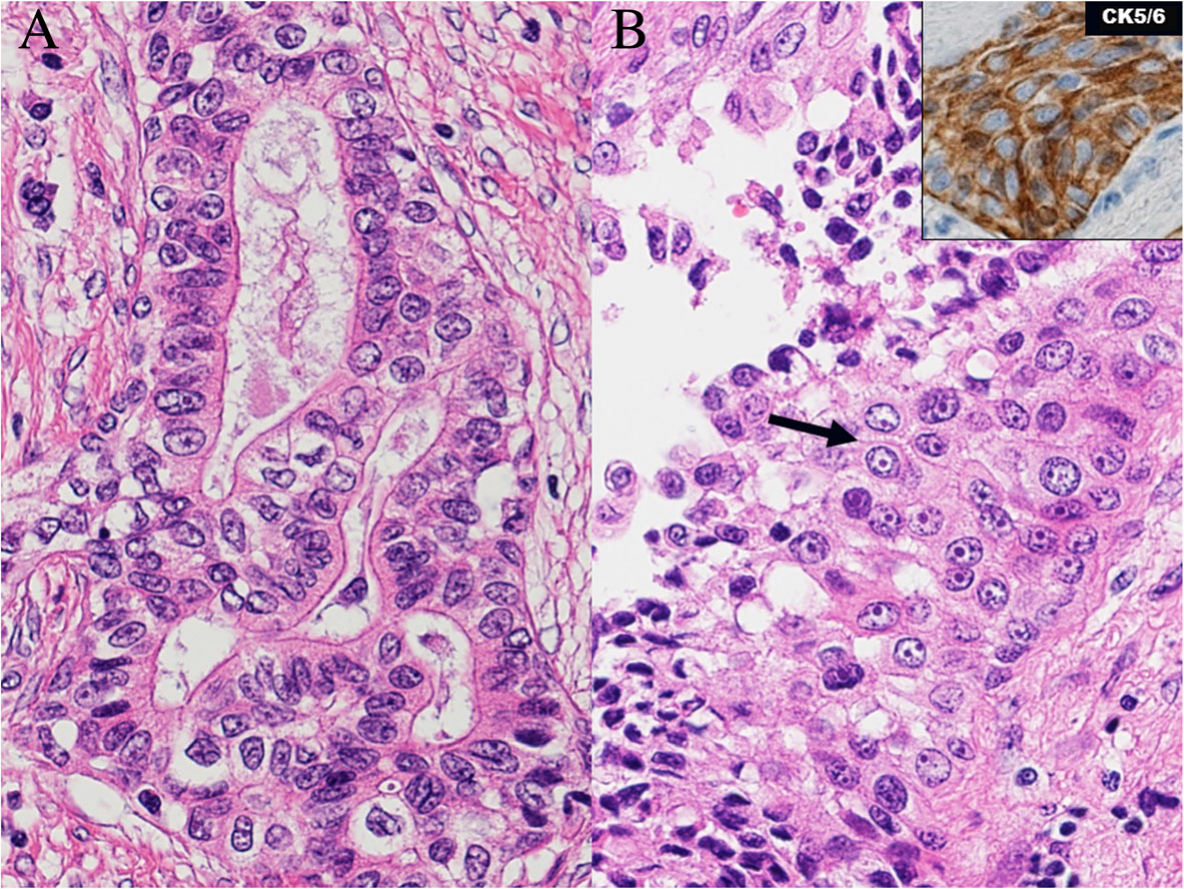
Hematoxylin and eosin stain of the primary lesion at necropsy. Approximately 20% of the lesion manifested several characteristics of squamous cell carcinoma as determined by the presence of intercellular bridges (*arrow*) and cytokeratin 5/6 positivity, indicating an adenosquamous carcinoma (**a**, **b**). *CK5/6* cytokeratin 5/6

After his death, we performed pathologic dissection with the consent of his family. Pathological autopsy showed severe cerebral edema and compression of the brain stem. As a result, respiratory arrest due to brain stem compression was considered to be the direct cause of death. In addition, the primary lesion in the left lung was re-evaluated histopathologically, and approximately 20% of the lesion manifested several characteristics of squamous cell carcinoma as determined by the presence of intercellular bridges and cytokeratin (CK) 5/6 positivity, indicating an adenosquamous carcinoma (Fig. 4a, b). Histological findings of leptomeningeal carcinomatous lesions also pointed to adenosquamous carcinoma, and the proportion of the squamous cell carcinoma component was higher than that of the left lung.

## Discussion

Alectinib is an ALK inhibitor with high selectivity for *EML4*-*ALK*, and in recent years a direct comparison trial with crizotinib demonstrated that its therapeutic effect was superior to that of crizotinib [5]. Also, the transitivity of alectinib to the brain is generally considered to be better, making it a drug that can be the first choice for cases of lung cancer with brain metastasis [6]. Based on pathological autopsy, the present case was ultimately diagnosed as *ALK*-positive lung adenosquamous carcinoma because carcinoma cells were immunohistologically positive for *ALK*. *ALK*-positive lung adenosquamous carcinoma is a rare cancer, with only a few cases being reported to date [7, 8]. There are no reports on the effect of ALK inhibitors on *ALK*-positive lung adenosquamous carcinoma; therefore, its clinical course is unknown. In general, lung adenosquamous carcinoma is known to have a poor prognosis as compared with adenocarcinoma and squamous cell carcinoma [9]. Although its sensitivity to ALK inhibitors is unknown, the fact that this was a case of adenosquamous carcinoma but not pure adenocarcinoma may be one reason for its aggressive nature.

In this case, the primary tumor and metastatic brain tumor were temporarily reduced by an ALK inhibitor, but leptomeningeal carcinomatosis developed only 3 months after initiation of treatment. At necropsy of the site exhibiting leptomeningeal carcinomatosis, the ratio of the squamous cell carcinoma component to the adenocarcinoma component was higher than that in the primary tumor of the left lung.

Regarding the prognosis of adenosquamous carcinoma, there is a report that the prognosis is worse for the tissue type with a greater squamous cell carcinoma component [10]. In this case, the existence of tumors with different cell ratios may have in part led to exacerbation in a short period of time. Based on the autopsy results, we reviewed the pathological tissue from the transbronchial lung biopsy at the time of clinical diagnosis. The tissue specimen for clinical diagnosis in this case was a papillary adenocarcinoma. However, when this tissue was immunostained, thyroid transcription factor 1 (TTF-1)-negative and CK5/6-positive parts were recognized. This result indicates that we could diagnose this patient as having adenosquamous carcinoma of the lung. Although the prognosis of lung cancer has improved owing to the development of current treatments, there are cases where the prognosis is poor, such as adenosquamous carcinoma. Pathological diagnosis of adenosquamous carcinoma is actually difficult; therefore, it is necessary to review the tumor composition when encountering cases of *ALK*-positive lung adenocarcinoma that respond poorly to ALK inhibitors.

## Conclusion

In summary, we reported an autopsy case of *ALK*-positive lung cancer that worsened in a short time. An autopsy revealed adenosquamous carcinoma of the lung, suggesting that it was involved in the prognosis of our patient. In cases of *ALK*-positive lung adenocarcinoma poorly responsive to treatment, re-examination of the tissue should be considered because there is a possibility of adenosquamous carcinoma.
